# Profile of time-dependent VEGF upregulation in human pulmonary endothelial cells, HPMEC-ST1.6R infected with DENV-1, -2, -3, and -4 viruses

**DOI:** 10.1186/1743-422X-6-49

**Published:** 2009-05-06

**Authors:** Azliyati Azizan, Kelly Fitzpatrick, Aimee Signorovitz, Richard Tanner, Heidi Hernandez, Lillian Stark, Mark Sweat

**Affiliations:** 1 Global Health Department, College of Public Health, 13201 Bruce B Downs Bvld, Tampa, Florida 33612, USA; 2 Florida Department of Health, Bureau of Laboratory, 3602 Spectrum Blvd, Tampa, Florida 33612, USA

## Abstract

In this study, the upregulated expression level of vascular endothelial growth factor (VEGF) in a pulmonary endothelial cell line (HPMEC-ST1.6R) infected with dengue virus serotypes 1, 2, 3, and 4 (DENV-1, -2, -3 and -4), was investigated. This cell line exhibits the major constitutive and inducible endothelial cell characteristics, as well as angiogenic response. Infection by all four DENV serotypes was confirmed by an observed cytopathic effect (CPE), as well as RT-PCR (reverse-transcription polymerase chain reaction) assays. As we had previously reported, the DENV-infected HPMEC-ST1.6R cells exhibited an elongated cytoplasmic morphology, possibly representing a response to VEGF and activation of angiogenesis. In this study, increase in VEGF expression level at designated time points of 0, 8, 24, 96 and 192 hours post-infection was investigated, using a microbead-based Bio-Plex immunoassay. Increased level of VEGF expression in infected-HPMEC-ST1.6R was detected at 8 hours post-infection. Interestingly, VEGF expression level began to decrease up to 96 hours post-infection, after which an upsurge of increased VEGF expression was detected at 192 hours post-infection. This profile of VEGF upregulated expression pattern associated with DENV infection appeared to be consistent among all four DENV-serotypes, and was not observed in mock-infected cells. In this study, the expression level of VEGF, a well-established vascular permeabilizing agent was shown to be elevated in a time-dependent manner, and exhibited a unique dual-response profile, in a DENV-infected endothelial cell. The experimental observation described here provided additional insights into potential mechanism for VEGF-mediated vascular leakage associated with DENV, and support the idea that there are potential applications of anti-VEGF therapeutic interventions for prevention of severe DENV infections.

## Findings

Dengue fever (DF) and dengue hemorrhagic fever (DHF) are caused by one of four closely related, but antigenically distinct, dengue virus serotypes 1, 2, 3 or 4 (DENV-1, DENV-2, DENV-3, and DENV-4) [1,2]. Several clinical manifestations including plasma leakage, thrombocytopenia and hemorrhage, distinguish DHF from DF, which is a milder infection [2-4]. Severe DHF, also known as dengue shock syndrome (DSS), can occur when fluid leakage into interstitial tissue spaces leads to hypovolemic shock. Plasma levels of various cytokines such as TNF-α, IFN-γ and IL-8, were found to be significantly higher in DHF patients, when compared to DF patients [5]. DENV infection of target cells *in-vitro*, induces increased expression level of these cytokines and other growth factors such as VEGF. Previous studies have inferred that endothelial cell damage may also be mediated through effects of induced VEGF expression in DENV-infected cells [5-8]. These perturbations are thought to at least be partly responsible for some of the clinical manifestations of hemorrhage and capillary leakage associated with DHF.

**Figure 1:**
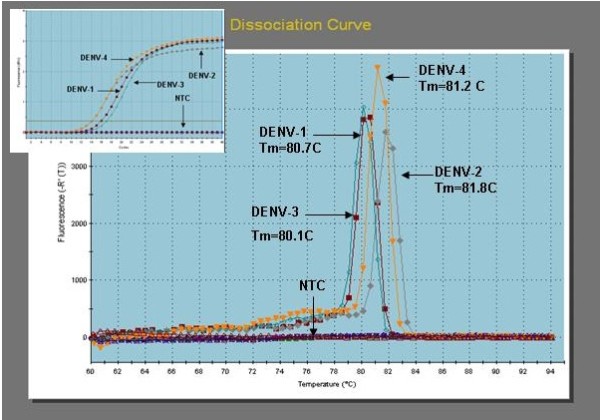
**RT-PCR analysis of DENV-1, -2, -3 and 4 infected HPMEC-ST1.6R cells**. Cell culture media of HPMEC-ST1.6R cells infected with DENV-1, -2, -3 and -4 were collected and used to obtain total RNA. A real-time RT-PCR analysis of HPMECST1.6R cells infected with DENV-1, -2, -3 and -4 were subsequently performed using the extracted RNA for quantitative analysis, using the MXPro3000P [Stratagene]. A SYBR Green 1 kit [Stratagene] and the DN-F/DN-R primer set [10] were utilized for amplifications, following instructions specified by the manufacturer. Following amplification, melting curve analysis was performed by raising the incubation temperature from 62°C to 95°C to verify correct amplification product by its specific melting temperature. DENV specific products derived from Vero or HPMEC cells infected with DENV-1 displayed a T_m _of 80.7°C, while DENV-2, -3 and -4 displayed specific products with T_m _values of 81.8°C, 80.1°C and 81.2°C, respectively.

The goal of this study was to quantify secreted VEGF level in an endothelial cell line that has been infected by all four serotypes of DENV viruses, at specific time points of 0, 8, 24, 96 and 192 hours post-infection. Human pulmonary microvascular endothelial cell (HPMEC-ST1.6R), an endothelial cell line generated in 2001, was found to exhibit most of the phenotypes associated with primary human microvascular endothelial cells [9]. These cells that were infected with DENV-1, -2, -3, and -4 viruses as we had previously described [6], showed cytopathic effect (CPE) starting from days 1 to 8 post-infection when compared to mock-infected cells (data not shown). The high titer DENV viruses used in this study were generated in VERO cells. Various forms of CPE were observed post-infection including cellular clumping, floating cells suggestive of apoptosis or necrosis, as well as elongated cellular morphology. In the latter scenario, elongated cells began to be observed at hour 24 post-infection, and some cells were seen at a later time point to detach into the cell medium. The floating and clumping CPE morphology started to form after 5 days post-infection. The infectivity of the DENV virions recovered from HPMEC-ST1.6R infected cells was evaluated using plaque formation assay, in which monolayers of vero cells were infected with 10-fold serial dilutions of the tissue culture supernanant, and the viral plaques were visualized using MTT staining (data not shown). A real-time RT-PCR analysis of HPMECST1.6R infected with DENV-1, -2, -3 and -4 were subsequently performed for quantitative analysis, using the MXPro3000P (Stratagene). A SYBR Green 1 kit [Stratagene] and the DN-F/DN-R primer set [10] were utilized for amplifications, following instructions specified by the manufacturer. The DN-F/DN-R primer set which could bind the templates from all four DENV serotypes, showed specific amplification (Fig. 1, inset) for all four infected samples. No amplicon was detected in negative control sample (no template control, NTC). Following amplification, melting curve analysis was performed by raising the incubation temperature from 62°C to 95°C to verify correct amplification product by its specific melting temperature. DENV specific products derived from HPMEC-ST1.6R cells infected with DENV-1 displayed a T_m _of 80.7°C, while DENV-2, -3 and -4 displayed specific products with T_m _values of 81.8°C, 80.1°C and 81.2°C, respectively (Fig. 1). The RT-PCR analyses provided further evidence that HPMEC-ST1.6R could be infected by all four DENV serotypes, as the RNA for these DENV viruses could be detected in infected cells.

**Figure 2:**
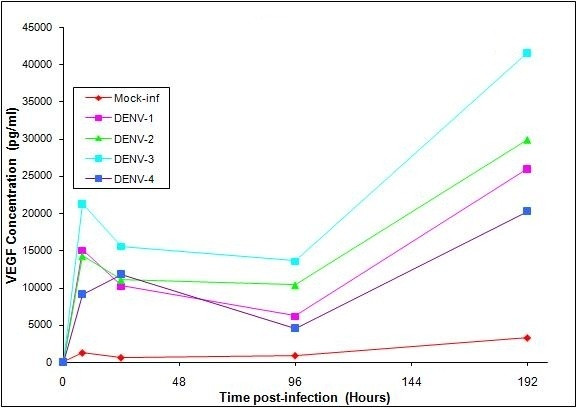
**VEGF Production in HPMEC-ST1.6R Cells infected with DENV-1, -2, -3, and -4**. Monolayers of HPMEC-ST1.6R cells were infected with either DENV-1, -2, -3 or DENV-4 viruses as previously described [6], and incubated at 37°C in 5% CO_2 _for 6 days. The cell media of DENV-infected cells and mock-infected cells were collected at 0, 8, 24, 96 and 192 hours post-infection, and analyzed for VEGF production [actual values listed in Table 1]. Mean VEGF levels were determined using a VEGF analyte detection kit and a BioPlex suspension array analyzer from BioRad. Significant increases (p ≤ 0.05) in cytokines between virus-infected and mock-infected cell cultures are given in Table 2. [Bars equal standard deviation from the mean of triplicates].

We reported in a previous study that DENV-infected HPMEC-ST1.6R showed increased levels of specific cytokines and VEGF, and suggested that the elongated cytoplasmic morphology in infected cells could be the result of VEGF-mediated activation of angiogenesis [6]. To further verify this observation, we quantified the VEGF levels in the supernatant of DENV-1, -2, -3 and -4 infected HPMEC-ST1.6R cells at specific time points of 0, 8, 24, 96 and 192 hours post-infection using a microsphere-based immunoassay which utilized Luminex™ beads coupled to VEGF-specific antibodies as an analyte capture platform (BioPlex, Biorad, Hercules, California, USA), essentially as we had previously described [6]. The reacted beads were analyzed on a Bio-Plex plate reader; assay controls consisted of beads which were not reacted with sample or standards, but otherwise treated as previously described. Standard curves and the concentration of cytokines within samples were generated through Bio-Plex Manager 4.0 software. Analysis of data was completed using five-parametric-curve fitting. Statistical analysis to compare infected and non-infected culture supernatants included Students-T test where differences were considered significant at P ≤ 0.05. DENV-infected cells showed a higher level of VEGF when compared to mock-infected cells (Table 1 and Fig. 2). An interesting display of profile consistent across all four DENV serotypes was observed, whereby VEGF level increased rapidly 8 hours post-infection and then hit a plateau at around 96 hours post-infection. Following this time point, the VEGF levels increased again, this time significantly up to 192 hours (8 days) post-infection. The Bio-Plex assay showed a significant (P ≤ 0.05) increase of VEGF in DENV-2-infected cells, when compared to mock-infected cells for all the time points analyzed (Table 2). The observed dual profile was represented by an initial primary increased expression level of VEGF, followed by a secondary increase in VEGF level. This could be due to, (1) a direct DENV infection giving rise to the primary effect of VEGF upregulation, which was then followed by, (2) the secondary VEGF increased level mediated by secreted cytokines, which included also VEGF. We attribute the secondary "burst" of VEGF upregulation to endothelial cells being in the "primed" state, which we propose took place after cells were being exposed to VEGF as well as other cytokines secreted immediately following the DENV-infection. The expression level of VEGF can be induced by other factors such as FGF-4, PDGF, TNF-α, TGF-β, insulin-like growth factor 1, IL-1β, IL-6 and PGE2 [11]. We and others have shown that expression of some of these cytokines become increased as a consequence of infection by DENV [5,6,12,13]; these upregulated cytokines then act on endothelial cells, placing these cells into the activated "primed" state which in turn induce secondary increased expression level of VEGF, as was observed in this study. We envision that in the *in-vivo *infection situation, the "primed" endothelial state would be greatly enhanced due to a cascade of cytokines secreted by other infected neighboring target cells such macrophages and dendritic cells [2,4,14], apart from the endothelial cells. This cytokine effects in turn could result in vascular leakage-associated immunopathologies associated with severe DHF diseases.

**Table 1:** VEGF levels (pg/ml) ± SD in cell culture conditioned medium of HPMEC-ST1.6R endothelial cells that were infected with DENV-1, -2. -3 and -4, or mock-infected [VM(-)] with vero cells culture medium.

Time (Hrs) Post-infection	VM (-) Mock	DENV-1 -infected	DENV-2 -infected	DENV-3 -infected	zDENV-4 -infected
0	0 ± 1.4	0 ± 5.7	0 ± 0.4	0 ± 1.4	0 ± 0

8	1,244.0 ± 1.4	14,997.5 ± 50.2	14,223.8 ± 460.3	21,275.6 ± 550.5	9,094.5 ± 220.6

24	600.1 ± 59.4	10,167.1 ± 384.3	11,039.2 ± 969.4	15,529.7 ± 238.3	11,782.0 ± 53.7

96	876.0 ± 28.6	6,150.6 ± 730.1	10,349.4 ± 189.9	13,541.0 ± 523.6	4,610.1 ± 207.9

192	3,247.7 ± 398.5	25,948.5 ± 432.0	29,889.6 ± 828.4	41,560.4 ± 131.2	20,281.7 ± 706.1

Samples were taken at time points 0, 8, 24, 96, and 192 hours.

**Table 2:** P-values for VEGF increased production levels comparing between DENV-infected and mock-infected HPMEC-ST1.6R endothelial cells.

Time (Hrs) Post-infection	DENV-1 -infected	DENV-2 -infected	DENV-3 -infected	DENV-4 -infected
0	0.542504	0.087579	0.167950	0.057191

8	0.000089	0.008074	0.006035	0.004234

24	0.008879	0.045426	0.001775	0.000297

96	0.079316	0.002276	0.010524	0.013508

192	0.006617	0.012808	0.001683	0.018874

Many studies have shown that vascular hyper-permeability which can lead to vascular leakage, can occur in response to a single, brief exposure in the endothelium to VEGF or other vascular permeabilizing agents [15]. VEGF, which is the most well-characterized pro-angiogenic growth factor, is involved not only in promoting angiogenesis which produces new blood vessels, but also in stimulating endothelial cell proliferation, migration, differentiation, tube formation, increased vascular permeability and maintaining vascular permeability [11]. Endothelial cells infected by another viral agent, Hepatitis C virus [16], and epithelial cells infected by human rhinovirus [17] were also shown to induce increased expression level of VEGF, implicating their roles in mediating immunopathologies associated with these specific viral infections. Hantaviruses which cause two lethal vascular permeability-based diseases; hemorrhagic fever with renal syndromes and hantavirus pulmonary syndromes [18] was reported to specifically enhance VEGF-directed permeabilizing responses in infected endothelial cells. This particular study implicated a direct role for VEGF in mediating vascular leakage and hemorrhagic diseases in these Hantaviruses-associated diseases and other vascular leakage syndromes.

Severity of plasma leakage in DENV patients was found to correlate with increased plasma levels of VEGF [8], but inversely correlated to soluble vascular endothelial growth factor receptor 2 (sVEGFR2) [7]. Interestingly, plasma viral load correlated well with a decline of VEGFR2, which is believed to bind to VEGF, controlling its availability and interfering with its cellular function. One proposed implication from this study was that VEGF participates in regulating vascular permeability that leads to plasma leakage seen in DHF patients, and that its activity and availability is controlled by a soluble form of its receptor, sVEGFR2. A related study which was cited earlier [18] showed that hantavirus-directed permeability in infected endothelial cell could be inhibited by antibodies to VEGFR2, which implicates its therapeutic potential in the treatment of vascular leakage and hemorrhagic diseases. We have shown in our study that the expression level of VEGF was elevated in a time-dependent manner, and exhibited a unique dual-response profile in endothelial cells infected by all four serotypes of DENV viruses. The experimental observation described here could provide insights into potential mechanism for VEGF-mediated vascular leakage associated with DENV. Findings from this and other related studies could provide impetus to further establish use of anti-VEGFR2 and other potential anti-VEGF agents [19-21] as therapeutic interventions for treatment and prevention of vascular leakage associated with DHF.

## Competing interests

The authors declare that they have no competing interests.

## Authors' contributions

AA designed the experiments, trained students in experimental protocols, maintained cell culture and performed DENV-infections, assisted in data analysis and wrote the manuscript, KF performed the microbead immunoassay, AS performed DENV infections, RT-PCR and assisted in data analysis and preparations of figures, RT optimized the RT-PCR conditions used in this study, HH optimized microbead immunoassay conditions used in this study, LS provided the laboratory facilities, expertise, advise and supervision to everyone involved in this study and critically reviewed the manuscript, and MS provided training, expertise and reagents for the microbead immunoassay.

## References

[B1] Halstead SB (2007). Dengue. Lancet.

[B2] Kurane I (2007). Dengue hemorrhagic fever with special emphasis on immunopathogenesis. Comp Immunol Microbiol Infect Dis.

[B3] Leong AS (2007). The pathology of dengue hemorrhagic fever. Semin Diagn Pathol.

[B4] Pang T, Cardosa MJ, Guzman MG (2007). Of cascades and perfect storms: the immunopathogenesis of dengue haemorrhagic fever-dengue shock syndrome (DHF/DSS). Immunol Cell Biol.

[B5] Basu A, Chaturvedi UC (2008). Vascular endothelium: the battlefield of dengue viruses. FEMS Immunol Med Microbiol.

[B6] Azizan A (2006). Differential proinflammatory and angiogenesis-specific cytokine production in human pulmonary endothelial cells, HPMEC-ST1.6R infected with dengue-2 and dengue-3 virus. J Virol Methods.

[B7] Srikiatkhachorn A (2007). Virus-induced decline in soluble vascular endothelial growth receptor 2 is associated with plasma leakage in dengue hemorrhagic Fever. J Virol.

[B8] Tseng CS (2005). Elevated levels of plasma VEGF in patients with dengue hemorrhagic fever. FEMS Immunol Med Microbiol.

[B9] Krump-Konvalinkova V (2001). Generation of human pulmonary microvascular endothelial cell lines. Lab Invest.

[B10] Shu PY (2003). Development of group- and serotype-specific one-step SYBR green I-based real-time reverse transcription-PCR assay for dengue virus. J Clin Microbiol.

[B11] Sivakumar R (2008). Kaposi's sarcoma-associated herpesvirus induces sustained levels of vascular endothelial growth factors A and C early during in vitro infection of human microvascular dermal endothelial cells: biological implications. J Virol.

[B12] Bosch I (2002). Increased production of interleukin-8 in primary human monocytes and in human epithelial and endothelial cell lines after dengue virus challenge. J Virol.

[B13] Suharti C (2002). The role of cytokines in activation of coagulation and fibrinolysis in dengue shock syndrome. Thromb Haemost.

[B14] Chaturvedi UC, Nagar R, Shrivastava R (2006). Macrophage and dengue virus: friend or foe?. Indian J Med Res.

[B15] Nagy JA (2008). Vascular permeability, vascular hyperpermeability and angiogenesis. Angiogenesis.

[B16] Kanda T (2008). Hepatitis C virus core protein augments androgen receptor-mediated signaling. J Virol.

[B17] Leigh R (2008). Human rhinovirus infection enhances airway epithelial cell production of growth factors involved in airway remodeling. J Allergy Clin Immunol.

[B18] Gavrilovskaya IN (2008). Hantaviruses direct endothelial cell permeability by sensitizing cells to the vascular permeability factor VEGF, while angiopoietin 1 and sphingosine 1-phosphate inhibit hantavirus-directed permeability. J Virol.

[B19] Edelman JL, Lutz D, Castro MR (2005). Corticosteroids inhibit VEGF-induced vascular leakage in a rabbit model of blood-retinal and blood-aqueous barrier breakdown. Exp Eye Res.

[B20] Pieramici DJ, Rabena MD (2008). Anti-VEGF therapy: comparison of current and future agents. Eye.

[B21] Sano H (2006). Negative regulation of VEGF-induced vascular leakage by blockade of angiotensin II type 1 receptor. Arterioscler Thromb Vasc Biol.

